# Multi-walled carbon nanotubes directly induce epithelial-mesenchymal transition in human bronchial epithelial cells via the TGF-β-mediated Akt/GSK-3β/SNAIL-1 signalling pathway

**DOI:** 10.1186/s12989-016-0138-4

**Published:** 2016-06-01

**Authors:** Manuela Polimeni, Giulia Rossana Gulino, Elena Gazzano, Joanna Kopecka, Arianna Marucco, Ivana Fenoglio, Federico Cesano, Luisa Campagnolo, Andrea Magrini, Antonio Pietroiusti, Dario Ghigo, Elisabetta Aldieri

**Affiliations:** 1 Department of Oncology, University of Turin, via Santena 5/bis, 10126 Turin, Italy; 2 Interdepartmental Centre Scansetti for Studies on Asbestos and Other Toxic Particulates, University of Turin, Turin, Italy; 3 Department of Chemistry, University of Turin, via Pietro Giuria 7, 10125 Turin, Italy; 4 NIS – Nanostructured Interfaces and Surfaces, University of Turin, via Pietro Giuria 7, 10125 Turin, Italy; 5 Department of Biomedicine and Prevention, University of Rome Tor Vergata, Via Montpellier 1, 00133 Rome, Italy

**Keywords:** Epithelial-mesenchymal transition, Lung fibrosis, Epithelial cells, Carbon nanotubes hazard, Fibrogenic potential, TGF-β, SNAIL-1 signalling pathway

## Abstract

**Background:**

Multi-walled carbon nanotubes (MWCNT) are currently under intense toxicological investigation due to concern on their potential health effects. Current in vitro and in vivo data indicate that MWCNT exposure is strongly associated with lung toxicity (inflammation, fibrosis, granuloma, cancer and airway injury) and their effects might be comparable to asbestos-induced carcinogenesis. Although fibrosis is a multi-origin disease, epithelial-mesenchymal transition (EMT) is recently recognized as an important pathway in cell transformation. It is known that MWCNT exposure induces EMT through the activation of the TGF-β/Smad signalling pathway thus promoting pulmonary fibrosis, but the molecular mechanisms involved are not fully understood. In the present work we propose a new mechanism involving a TGF-β-mediated signalling pathway.

**Methods:**

Human bronchial epithelial cells were incubated with two different MWCNT samples at various concentrations for up to 96 h and several markers of EMT were investigated. Quantitative real time PCR, western blot, immunofluorescent staining and gelatin zymographies were performed to detect the marker protein alterations. ELISA was performed to evaluate TGF-β production. Experiments with neutralizing anti-TGF-β antibody, specific inhibitors of GSK-3β and Akt and siRNA were carried out in order to confirm their involvement in MWCNT-induced EMT. In vivo experiments of pharyngeal aspiration in C57BL/6 mice were also performed. Data were analyzed by a one-way ANOVA with Tukey’s post-hoc test.

**Results:**

Fully characterized MWCNT (mean length < 5 μm) are able to induce EMT in an in vitro human model (BEAS-2B cells) after long-term incubation at sub-cytotoxic concentrations. MWCNT stimulate TGF-β secretion, Akt activation and GSK-3β inhibition, which induces nuclear accumulation of SNAIL-1 and its transcriptional activity, thus contributing to switch on the EMT program. Moreover, a significant increment of nuclear β-catenin - due to E-cadherin repression and following translocation to nucleus - likely reinforces signalling for EMT promotion. In vivo results supported the occurrence of pulmonary fibrosis following MWCNT exposure.

**Conclusions:**

We demonstrate a new molecular mechanism of MWCNT-mediated EMT, which is Smad-independent and involves TGF-β and its intracellular effectors Akt/GSK-3β that activate the SNAIL-1 signalling pathway. This finding suggests potential novel targets in the development of therapeutic and preventive approaches.

**Electronic supplementary material:**

The online version of this article (doi:10.1186/s12989-016-0138-4) contains supplementary material, which is available to authorized users.

## Background

Carbon nanotubes (CNT) are fibre-shaped promising and revolutionary nanomaterials that could have a wide range of applications due to their remarkable properties [1]. Given their potential, it is expected that the production and use of CNT will continue to grow over the coming years [2], raising concern on their potential health effects. Structurally, CNT can appear as single-walled (SWCNT) or multi-walled (MWCNT), which are thought to have the greatest potential health risks due to their reduced flexibility, high degree of biopersistence and similarity in shape to asbestos fibres [3].

Accumulating evidence in the literature shows the potential toxicity of MWCNT in biological systems and has demonstrated that MWCNT can induce lung inflammation, fibrosis, granuloma formation, cancer and airway injury in rodents [4–14]. MWCNT are also able to induce markers of remodelling and fibrosis in human in vitro models although no relationship of human diseases with the occupational or environmental exposure to MWCNT has been demonstrated so far. Snyder et al. observed altered morphology and loss of barrier function in bronchial epithelium exposed to non-cytotoxic doses of MWCNT [15], and Rotoli et al. detected changing of shape and cytoskeletal organization of several human epithelial cell lines exposed to MWCNT [16]. Hussain and colleagues demonstrated inflammasome activation in primary bronchial epithelial cells and subsequent markers of fibrosis in lung fibroblasts [17]. Some studies mainly focused on the role of lung interstitial fibroblasts as the primary responsive cells in pulmonary fibrosis: indeed, MWCNT may directly stimulate fibroblasts to produce collagen [18] or indirectly by stimulating macrophages to produce inflammatory cytokines [6, 10, 19]. Meanwhile, few papers investigated the function of epithelial cells in MWCNT-induced lung fibrosis. Kumarathasan and Ju studies evaluated the MWCNT cytotoxicity on human lung epithelial cells [20, 21]. Chen and colleagues suggested that epithelial cells could serve as a novel source of fibroblasts in pulmonary fibrosis through epithelial-mesenchymal transition (EMT) [22]: they demonstrated that MWCNT exposure induced pulmonary fibrosis and epithelial-derived fibroblasts via transforming growth factor-beta (TGF-β), one of the most potent EMT mediators [23, 24].

EMT is controlled by several mechanisms, however there is growing evidence that MWCNT exposure stimulates TGF-β production in fibroblasts [10, 19], macrophages [6] and epithelial cells [18, 22]. Recent studies finally showed that MWCNT are involved in the fibrogenic process by stimulating the TGF-β/Smad signalling pathway. Interestingly, it seems that the fibrotic response could depend on MWCNT length: only long MWCNT, but not short MWCNT, caused pulmonary injury and lung fibrosis promoted by the EMT and TGF-β/Smad signalling pathway [10, 19, 22]. On the other hand alveolar septal fibrosis in mice was induced only by exposure to short MWCNT [25]. Thus, the implication of CNT length in determining the fibrotic activity of MWCNT still needs to be confirmed by further investigations.

In light of the high concern about CNT potential hazard, we investigated in this work the occurrence of EMT in human bronchial epithelial (BEAS-2B) cells exposed to sub-cytotoxic concentrations of a sample of MWCNT that has been ground to reduce the length (MWCNTg; mean length < 5 μm) that we have previously studied [26]. Pristine MWCNT (pMWCNT) were tested for comparison. Moreover, we validated in vitro data in C57BL/6 mice. Interestingly, our results show that MWCNTg trigger the TGF-β-mediated SNAIL-1 signalling pathway, thus suggesting a novel mechanism of MWCNT-induced fibrosis and providing new important insights into cellular and molecular mechanisms involved.

## Results

### Chemical characterization of MWCNT

The pristine MWCNT (pMWCNT) is composed by low defective long and needle-like MWCNT (transmission electron microscopy, TEM, Additional file 1: Figure S1) having a wide range of lengths. As shown by scanning electron microscopy (SEM) micrographs (Additional file 1: Figure S1) several tubes appear to be longer than 10 μm albeit a quantitative measurement of length distribution is unfeasible. The sample contains iron and other impurities likely deriving from the catalyst used during the synthesis. Such physico-chemical properties are in reasonable agreement to what found by other authors on a material by the same company (MITSUI-7) [5]. When ground, the length distribution appeared more uniform, and tube longer than 5 μm were not found. Based on the analyses previously performed [27, 28], the mean length of MWCNTg was 1.12 μm and the mean diameter was 65–70 nm (see Table 1 for more detailed physico-chemical properties). At the same time, the grinding process decreased the crystallinity degree of the MWCNT, evaluated by Raman spectroscopy (Additional file 2: Figure S2), due to the insertion of defects following the cleavage of the C-C bonds.

**Table 1:** Physicochemical properties of MWCNT samples [26]

Samples	Mean diameter (nm)	Mean length (μm)	SSA (m^2^/g)	Elemental analysis (% oxides)	Iron potentially bioavailable (% Fe_2_O_3_)
pMWCNT	nd	nd	35.0	Fe 0.61 ± 0.01 Al 0.04 ± 0.05	0.50 ± 0.01
MWCNTg	67 ± 2	1.12 ± 0.05	60.3	Fe 0.50 ± 0.01 Al 0.06 ± 0.03	0.420 ± 0.001
Fe-depleted MWCNTg	70 ± 2	1.23 ± 0.05	52.6	Fe 0.050 ± 0.001 Al 0.06 ± 0.03	0.002 ± 0.001

For in vitro testing the MWCNT samples were dispersed by mild sonication. The degree of dispersion and the stability of the MWCNTg suspensions were evaluated by means of dynamic light scattering (DLS) and optical microscopy (OM): a peak, corresponding to agglomerates having an equivalent hydrodynamic diameter of 345 nm, was detected. However, OM revealed the presence of aggregates having a diameter of 1–5 μm. The polydispersion index (PDI) was 0.35, indicating a wide agglomerate size distribution (Additional file 3: Figure S3). No changes were detectable in either form or position of the peak after 15 min. After 24 h of incubation in cell medium a small but significant decrease of the mean equivalent hydrodynamic diameter evaluated by DLS and, at the same time, the formation of some large aggregates with a diameter around 10 μm was observed by OM. No significant changes were observed from 24 to 96 h (Additional file 3: Figure S3).

### Evaluation of cytotoxicity and cellular morphology in BEAS-2B cells exposed to MWCNT

**Fig. 1:**
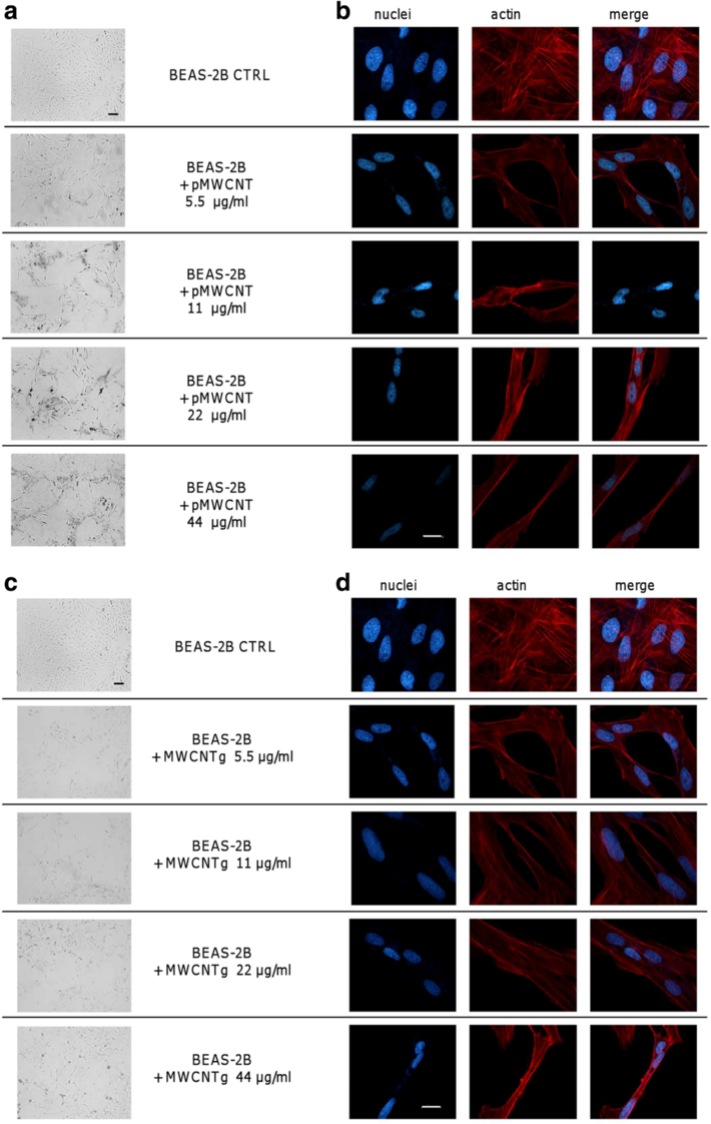
Representative microscope images of BEAS-2B cells. Cells were incubated for 96 h in either the absence (CTRL) or presence of different concentrations (5.5, 11, 22 and 44 μg/ml) of pMWCNT (**a**-**b**) or MWCNTg (**c**-**d**). After the incubation, the cells were rinsed with PBS and observed by optical microscopy (**a**-**c**) or fixed with paraformaldehyde and fluorescently labelled as described in Methods (**b**-**d**). **a**-**c** Representative images are shown (10×; scale bar = 50 μm). **b**-**d** Actin filaments were visualized in red, nuclei in blue. Representative images are shown (63×; scale bar = 10 μm)

Various concentrations of MWCNTg (5.5, 11, 22 and 44 μg/ml) incubated for different time periods (24, 48, 72 and 96 h) induced in BEAS-2B cells a slight increase of leakage of LDH activity into the extracellular medium (measured as ratio between extracellular and total LDH), taken as an index of augmented cell membrane permeability and cytotoxicity. After 96 h the LDH release was significantly increased only at the two highest concentrations: 14.9 % in control cells vs 29.2 and 31.9 % of cells exposed to 22 and 44 μg/ml of MWCNTg respectively. A similar behaviour was observed with pMWCNT (Additional file 4: Figure S4). In the same experimental conditions both optical images and fluorescence microscopy pictures demonstrated that, after long-term MWCNT exposure, most cells started to disorganize losing cellular junctions and their polygonal and regular shape. Indeed, after 96 h the cells exposed to both MWCNT samples had completely lost their organization in compact islets, taking on a tapered and spindle shape with pointed ends and elongated protrusions, assuming a fibroblast-like appearance with cells arranged in parallel, whereas control cells reached confluence and showed their typical epithelial morphology (Fig. 1). After these preliminary experiments we chose to perform 96 h-incubations in the absence or presence of sub-cytotoxic concentrations of MWCNT.

### Evaluation of epithelial and mesenchymal markers in BEAS-2B cells exposed to MWCNT

**Fig. 2:**
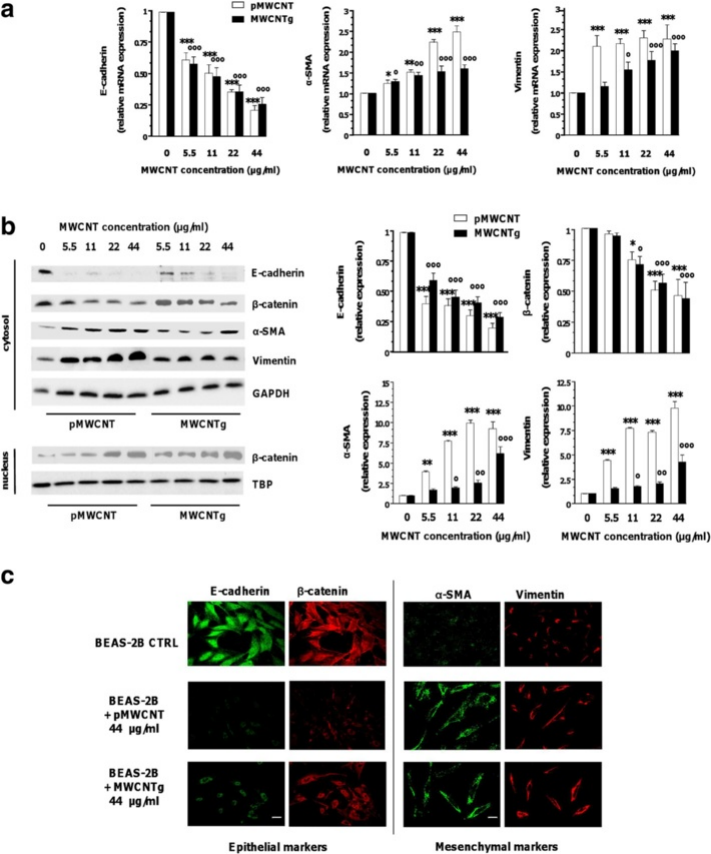
Relative expression of epithelial and mesenchymal markers of BEAS-2B cells exposed to MWCNT. Relative expression of epithelial and mesenchymal markers were checked by qRT-PCR (**a**), WB (**b**), confocal microscopy (**c**) in BEAS-2B cells incubated for 96 h in either the absence (0 μg/ml, control) or presence of different concentrations of pMWCNT or MWCNTg. **a** Relative expression of E-cadherin, α-SMA and vimentin mRNA. Results were expressed in units of relative mRNA expression compared to control cells (*n* = 3; white bar = pMWCNT; black bar = MWCNTg). Versus control *p < 0.05, **p < 0.001, ***p < 0.0001; Versus control °*p* < 0.05, °°*p* < 0.001, °°°*p* < 0.0001. **b** Relative expression of E-cadherin, β-catenin, α-SMA and vimentin proteins. GAPDH and TBP were used as loading control for cytosolic and nuclear extracts respectively. Results were expressed in units of relative protein expression compared to control cells (white bar = pMWCNT; black bar = MWCNTg). Each figure is representative of three experiments giving similar results. Versus control *p < 0.05, **p < 0.001, ***p < 0.0001; Versus control °*p* < 0.05, °°*p* < 0.001, °°°*p* < 0.0001. **c** After the incubation in the absence (0 μg/ml, CTRL) or presence of 44 μg/ml MWCNT, the cells were rinsed with PBS, fixed with paraformaldehyde and probed as described in Methods. E-cadherin and α-SMA were visualized in green, β-catenin and vimentin in red. Representative images are shown (60×; scale bar = 10 μm)

To evaluate if the EMT could be a potential mechanism behind this transformation, we examined the expression of classic epithelial and mesenchymal markers using quantitative real-time polymerase chain reaction (qRT-PCR) and Western blotting (WB). As shown in Fig. 2a, qRT-PCR data indicated that both MWCNT samples significantly enhanced the messenger RNA (mRNA) expression of the mesenchymal markers vimentin and α-smooth muscle actin (α-SMA), in a concentration-dependent manner. The effect was observed with both MWCNT samples, but was more evident with the pristine one. At the same time the mRNA expression of E-cadherin – a typical epithelial protein of cellular adherens junctions - was significantly repressed with both MWCNT samples (no change was observed in β-catenin mRNA, not shown). WB showed significantly increased expression of vimentin and α-SMA, more marked with pMWCNT, and loss of E-cadherin in response to exposure to both MWCNT (Fig. 2b). In addition, we observed a significantly reduced expression of cytosolic β-catenin, an essential partner of E-cadherin in cell-cell adhesion complexes, associated to an enhanced nuclear accumulation of the protein (Fig. 2b). The MWCNT-induced alteration of protein expression was further confirmed by immunofluorescence. After exposure of BEAS-2B cells to both MWCNT samples (44 μg/ml) for 96 h, E-cadherin and β-catenin staining decreased, whereas vimentin and α-SMA increased, thus confirming the immunoblot data. Moreover, confocal immunofluorescence microscopy clearly showed that α-SMA – a member of actin family typically expressed by fibroblasts – and the intermediate filament vimentin expression was enhanced patching at the cell membrane, thus confirming the cytoskeleton reorganization and altered cell morphology (Fig. 2c).

### Evaluation of extracellular proteins in BEAS-2B cells exposed to MWCNT

**Fig. 3:**
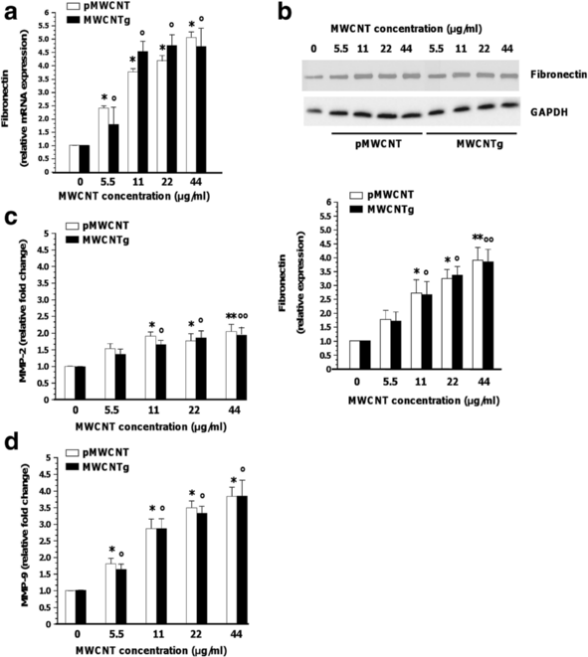
Relative expression of fibronectin (**a**-**b**) and metalloproteinases activity (**c**-**d**) in BEAS-2B cells exposed to MWCNT. Cells were incubated for 96 h in either the absence (0 μg/ml, control) or presence of pMWCNT or MWCNTg at the concentration of 5.5, 11, 22 and 44 μg/ml. **a** Relative expression of fibronectin mRNA checked by qRT-PCR. Results were expressed in units of relative mRNA expression compared to control cells (*n* = 3; white bar = pMWCNT; black bar = MWCNTg). Versus control *p < 0.0001; Versus control °*p* < 0.0001. **b** Relative expression of fibronectin protein checked by WB. Results were expressed in units of relative protein expression compared to control cells (white bar = pMWCNT; black bar = MWCNTg). Each figure is representative of three experiments giving similar results. Versus control *p < 0.05, **p < 0.0001; Versus control °*p* < 0.05, °°*p* < 0.0001. **c**-**d** Metalloproteinases activity checked by zymography. Results were expressed as relative fold change compared to control cells (white bar = pMWCNT; black bar = MWCNTg) (*n* = 3). **c** Versus control *p < 0.001, **p < 0.0001; Versus control °*p* < 0.001, °°*p* < 0.0001. **d** Versus control *p < 0.0001; Versus control °*p* < 0.0001

The up-regulation of genes encoding extracellular proteins, such as the extracellular matrix (ECM) protein fibronectin and metalloproteinases (MMPs), has been recognized as main hallmark of EMT and fibrosis [29, 30]. The MWCNT treatment caused a similar significant and concentration-dependent increment of fibronectin mRNA expression (Fig. 3a) in cells exposed to both MWCNT samples. BEAS-2B cells also showed increased extracellular fibronectin levels (Fig. 3b): fibronectin is an integral constituent of the fibrotic ECM, therefore it is likely that the protein is produced and extruded out of the cell. However, it is interesting to highlight that the total amount of fibronectin was significantly enhanced and the increment was concentration-dependent. MMPs are a family of neutral proteinases, well known for their ability to degrade and remodel ECM proteins, an event that in epithelial cells associates with EMT processes. Our data showed a significantly raised activity of MMP-2 and MMP-9 in the extracellular medium of cells exposed for 96 h to increasing concentrations of both MWCNT samples (Fig. 3c, d).

### Evaluation of cellular morphology and markers expression in BEAS-2B cells exposed to Fe-depleted MWCNTg

CNT toxicity is generally attributed to the residual metals used as catalysts in the production process. Therefore, in order to discriminate the biological effect of MWCNTg alone, BEAS-2B cells were incubated for 96 h with a concentration of Fe^3+^ similar to the one potentially released at the highest concentration of MWCNTg used and to Fe-depleted MWCNTg (44 μg/ml see Table 1). Data showed that cell morphology and epithelial markers are not modified by Fe^3+^ alone. In addition Fe-depleted MWCNTg, prepared by acidic treatment of MWCNTg [26], were able to induce in BEAS-2B cells morphological changes and altered proteins expression similar to that induced by MWCNTg (Additional file 5: Figure S5).

### Evaluation of Akt/GSK-3β/SNAIL-1 signalling pathway in BEAS-2B cells exposed to MWCNT

**Fig. 4:**
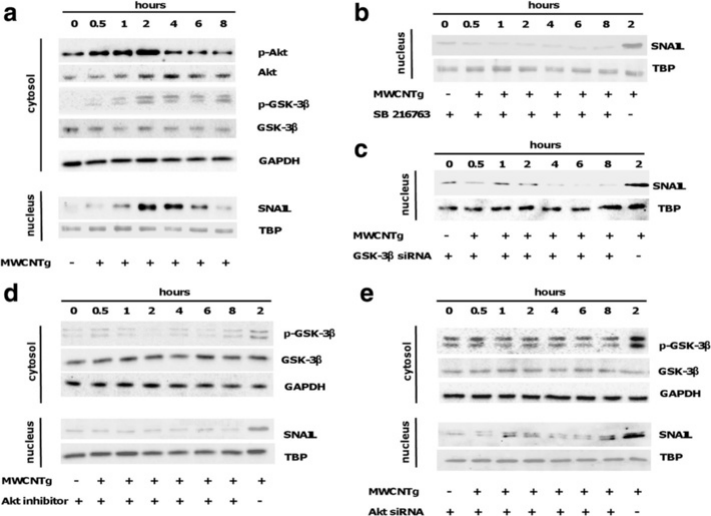
Akt/GSK-3β/SNAIL-1 signalling pathway in BEAS-2B cells exposed to MWCNTg. Cells were incubated in either the absence (0 μg/ml, control) or presence of 44 μg/ml of MWCNTg from 30 min to 8 h (**a**), together with the specific GSK-3β inhibitor SB216763 (**b**), GSK-3β siRNA (**c**), the specific Akt 1/2 Kinase Inhibitor (**d**) or Akt 1/2 siRNA (**e**). Each figure is representative of three experiments giving similar results

**Fig. 5:**
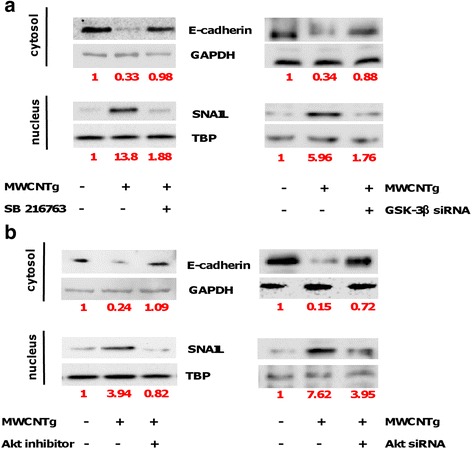
Akt and GSK-3β inhibition effect in BEAS-2B cells exposed to MWCNTg. The cells were incubated in either the absence (0 μg/ml, control) or presence of 44 μg/ml of MWCNTg for 96 h, without and with 5 μM SB216763 or 10 nM GSK-3β siRNA (**a**), without and with 5 μM of the specific Akt 1/2 Kinase Inhibitor or 50 nM Akt 1/2 siRNA (**b**). Each figure is representative of three experiments giving similar results. Results were expressed in units of relative protein expression compared to control cells (indicated in red in the figure)

It has been demonstrated that MWCNT can induce the canonical TGF-β signaling pathway [10, 19] thus leading to the activation of the Smad proteins. However, TGF-β could also induce the activation of the Smad-independent pathway that has SNAIL-1 as a main downstream transcription factor [23, 24, 31–33]. SNAIL-1 is negatively regulated by protein glycogen synthase kinase-3β (GSK-3β), an ubiquitously expressed serine/threonine kinase [34], crucial to stabilize SNAIL-1 [35, 36]. The pathway GSK/SNAIL is known to regulate EMT [32]. Therefore, to further investigate the mechanism leading to the decrease of E-cadherin expression, we evaluated the involvement of SNAIL-1 transcriptional activity and GSK-3β [35, 36]. We performed time-dependent experiments in which BEAS-2B cells were incubated with 44 μg/ml MWCNTg up to 8 h. This concentration for this incubation time did not exert a cytotoxic effect (data not shown). The results showed significant SNAIL-1 nuclear accumulation after 1 h of exposure to MWCNTg, with a significant corresponding increment of cytosolic GSK-3β phosphorylation (p-GSK-3β) (Fig. 4a). After 4 h of incubation the nuclear levels of SNAIL-1 decreased. To confirm the role of GSK-3β we incubated BEAS-2B cells with MWCNTg and 5 μM SB216763, a specific inhibitor of GSK-3β, or with MWCNTg and 10 nM GSK-3β siRNA and in both cases no increase of SNAIL-1 in the nucleus was observed (Fig. 4b, c). GSK-3β phosphorylation could result from the activation of the Smad-independent pathway of TGF-β signalling by recruiting protein kinase B (PKB)/Akt as an upstream kinase for GSK-3β [23, 24, 31–33, 37]. Thus, we next evaluated the phosphorylated Akt (p-Akt) levels in BEAS-2B cells exposed to 44 μg/ml of MWCNTg up to 8 h. Our data demonstrated a significant increment of p-Akt after 30 min, reaching a maximum at 2 h: its phosphorylation returned similar to control levels at 4–6 h (Fig. 4a). The recruitment of Akt in this pathway was confirmed by incubating MWCNTg-exposed BEAS-2B cells with 5 μM of the specific Akt 1/2 Kinase Inhibitor or 50 nM Akt 1/2 siRNA: the Akt inhibition kept unaltered both cytosolic p-GSK-3β and nuclear accumulation of SNAIL-1 compared to control cells (Fig. 4d, e). Moreover, after a 96 h-incubation of BEAS-2B cells with 44 μg/ml of MWCNTg together with Akt and GSK-3β siRNA or specific inhibitors (SB216763 or Akt inhibitor), the WB analysis showed that, while the treatment with MWCNT increased SNAIL-1 accumulation in the nucleus and inhibited the expression of E-cadherin, the co-incubation with the siRNA genes knockdown or inhibitors reversed completely the nuclear accumulation of SNAIL-1 and partially restored the control levels of E-cadherin (Fig. 5a, b).

### Evaluation of TGF-β production in BEAS-2B cells exposed to MWCNT

**Fig. 6:**
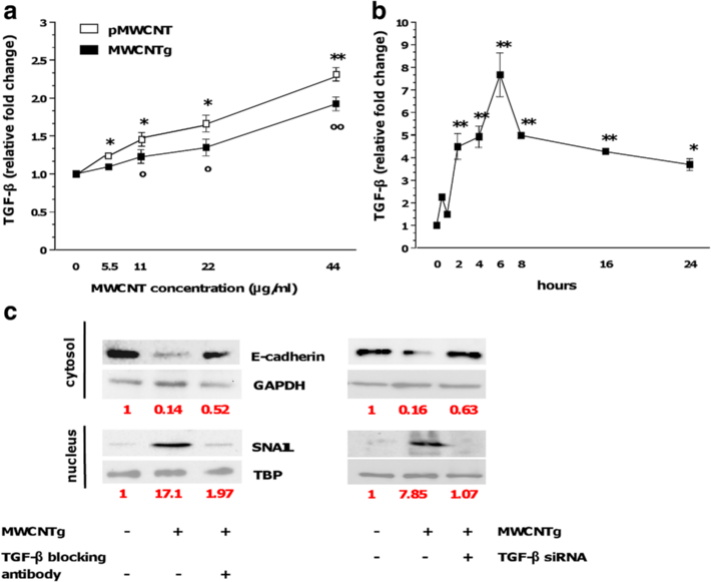
TGF-β production (**a**-**b**) and TGF-β inhibition effect in BEAS-2B cells exposed to MWCNT (**c**). Cells were incubated for 96 h in either the absence (0 μg/ml, control) or presence of pMWCNT (white square) or MWCNTg (black square) at the concentration of 5.5, 11, 22 and 44 μg/ml (**a**) or incubated up to 24 h in either the absence (0 μg/ml, control) or presence of 44 μg/ml MWCNTg (**b**). The TGF-β production was evaluated by ELISA in the supernatants of cells exposed to MWCNT. Results were expressed as relative fold change compared to control cells (*n* = 3). No significant variation was observed in control cells at the different time points evaluated. **a** Versus control *p < 0.05, **p < 0.0001; Versus control °*p* < 0.05, °°*p* < 0.0001. **b** Versus control *p < 0.05, **p < 0.0001. **c** The cells were incubated in either the absence (0 μg/ml, control) or presence of 44 μg/ml of MWCNTg for 96 h, without and with 5 μg/ml of neutralizing anti-TGF-β antibody or 10 nM TGF-β siRNA. The efficacy of TGF-β siRNA was evaluated by semi-quantitative PCR. Each figure is representative of three experiments giving similar results. Results were expressed in units of relative protein expression compared to control cells (indicated in red in the figure)

Among the mediators involved in EMT, TGF-β is considered a critical stimulus [23, 24, 31–33]. The TGF-β production was assessed with an ELISA kit in the supernatant of BEAS-2B cells exposed to different concentrations of both MWCNT samples. As depicted in Fig. 6a, in agreement with literature our data showed that TGF-β levels significantly increased after 96 h in a concentration-dependent manner and the increment was a little marked with pMWCNT. Since the previously investigated signalling pathways are activated at shorter times, we next analyzed the TGF-β production in BEAS-2B cells exposed to 44 μg/ml of MWCNTg up to 24 h. Results revealed a significant rapid increment of TGF-β secretion starting after 2 h of MWCNTg exposure (corresponding to the activation of p-Akt previously described) and reaching a maximum after 6 h; then TGF-β production slightly decreased, although the increment was still significant at 24 h (Fig. 6b). Finally, in order to confirm whether TGF-β could be a mediator of the observed EMT, a neutralizing anti-TGF-β antibody and TGF-β siRNA gene knockdown were used. The co-incubation for 96 h of BEAS-2B cells with 44 μg/ml of MWCNTg and 5 μg/ml of TGF-β-blocking antibody or with 10 nM TGF-β siRNA gene knockdown partially recovered the control levels of E-cadherin and completely reversed the SNAIL-1 nuclear accumulation (Fig. 6c). In parallel, no cellular morphological alterations were observed (not shown).

### Evaluation of TGF-β production and development of lung fibrosis in C57BL/6 mice exposed to MWCNT

**Fig. 7:**
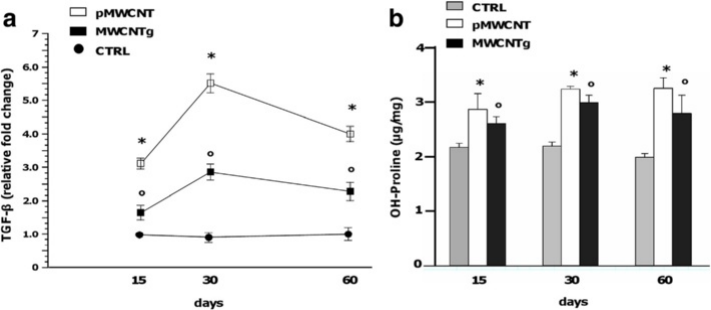
TGF-β production (**a**) and lung hydroxyproline content (**b**) in C57BL/6 mice exposed to MWCNT. **a** C57BL/6 mice were exposed to sterile water (control, CTRL, black circle) or 120 μg of pMWCNT (white square) or MWCNTg (black square) for 15, 30 and 60 days. At the end BAL were collected and used for TGF-β measurement by ELISA. Versus control *p < 0.0001; Versus control °*p* < 0.0001. **b** Content of hydroxyproline measured in lung tissue of animals exposed to pMWCNT (white bars) and MWCNTg (black bars) compared to controls (CTRL, grey bars). Versus control *p < 0.05; Versus control °*p* < 0.05

**Fig. 8:**
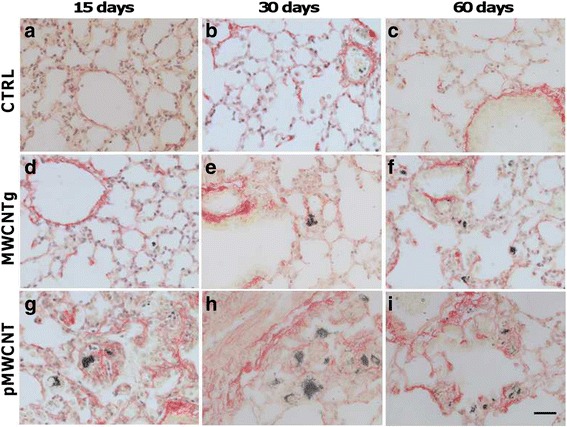
Representative microscope images of collagen deposition in lungs. Sirius red staining of lung sections from control animals (CTRL, panels **a**, **b**, **c**), animals exposed to MWCNTg (**d**, **e**, **f**) and to pMWCNT (**g**, **h**, **i**) at 15, 30 and 60 days. Aggregates of the nanotubes are clearly visible as black spots on the sections, red staining represents collagen deposition. Representative images are shown (40×; scale bar = 30 μm)

In vivo experiments of pharyngeal aspiration in C57BL/6 mice were performed to support the in vitro results. We measured the production of TGF-β in the broncho-alveolar lavage (BAL) of mice exposed to 120 μg of pMWCNT and MWCNTg after 15, 30 and 60 days. pMWCNT were able to induce a significant increment of TGF-β release in BAL already after 15 days from the exposure; the increment reached a maximum after 30 days and then decreased, although it was still significant after 60 days (Fig. 7a). TGF-β levels significantly increased also in mice exposed to MWCNTg: the increment showed a similar behaviour, reaching its maximum after 30 days and then decreasing, although the production was less evident (Fig. 7a). In addition, we collected lung tissues from both MWCNT-exposed mice and evaluated the development of fibrosis through hydroxyproline dosage and Sirius red staining. Interestingly, the dosage of hydroxyproline lung content clearly demonstrated a statistically significant higher deposition of collagen in the lungs of animals treated with both MWCNT samples (Fig. 7b). Similarly, histological analysis of Sirius red-stained lung sections demonstrated increased deposition of collagen in animals exposed to both MWCNT samples, although the staining was more pronounced in the sections of pMWCNT-exposed mice (Fig. 8).

**Fig. 9:**
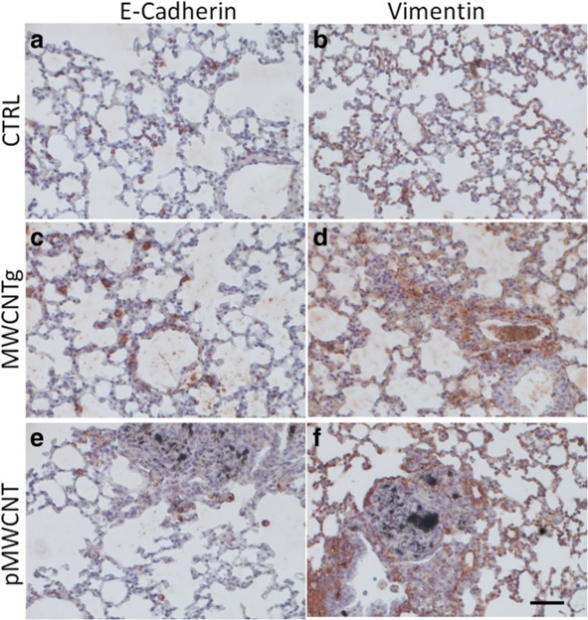
Representative immunoistochemical images of E-cadherin and vimentin in lungs. Representative immunoistochemical images of E-cadherin and vimentin in lung sections from mice exposed at 30 days to MWCNTs through pharyngeal aspiration. E-cadherin was mainly expressed in type II pneumocytes in both control and MWCNT exposed lungs. The number of E-cadherin positive cells in NP-exposed compared to control sections was decreased (**a**, **c**, **e**), while the expression for vimentin was increased (**b**, **d**, **f**) (20×; scale bar = 60 μm)

We evaluated the expression of E-cadherin and vimentin on lung sections obtained from mice exposed to MWCNTs by pharyngeal aspiration 30 days before the analysis. Immunostaining of normal lung tissues revealed a predominant staining of type II pneumocytes as previously described (Fig. 9a) [38, 39]. Expression of E-cadherin was still visible in type II pneuomocyts of lungs from the MWCNT exposed mice, although the number of positive cells appeared decreased, particularly in sections from pMWCNT exposed lungs (Fig. 9c and e). At converse, the expression of vimentin appeared increased in the lung sections from animals exposed to the nanoparticles, compared to the control (Fig. 9b, d and f).

## Discussion

Since CNT have many industrial applications due to their unique properties [1], their raising production and use have promoted studies to investigate their potential adverse health effects following occupational or environmental exposure. Data in literature are growing, although results are not easily comparable because many parameters and characteristics may vary considerably. Current data, obtained in limited animal models in vivo and in human cells in vitro, indicate that CNT exposure could be strongly associated with lung toxicity with its effects being comparable to asbestos-induced carcinogenesis [3]. A large body of literature shows that CNT can enter cells accumulating and causing cell death: they may induce inflammation and oxidative stress [11, 40–42], exert genotoxic effects [43], lung cancer [14], mesothelioma [13], penetrate through lung tissues inducing fibrotic lesions [4–8, 10, 12, 44–46] and are able to migrate causing lesions in remote areas [47–49].

During pulmonary fibrosis, activation of macrophages and fibroblasts are common cellular events promoting the secretion of different inflammatory and fibrogenic factors, fibroblasts transformation for matrix deposition and remodelling [6, 8, 10, 50]. Recently, EMT has been increasingly recognized as an important pathway in fibrosis: differentiated epithelial cells undergo transition to a mesenchymal phenotype, giving rise to fibroblasts and myofibroblasts generation [51]. The EMT event was observed in vitro by Chen in human A549 cells [22] and by Wang in rat alveolar type-II epithelial cells [19] exposed to MWCNT. We carried out this study on human BEAS-2B cells, which are more sensitive than A549 cells [18], using two different MWCNT samples that we have previously characterized [26]. There are still some uncertainties about the MWCNT fibrotic potential due to the variety of results obtained. Pristine CNT are highly hydrophobic and the formation of agglomerates in aqueous solutions or biological fluids is inevitable. The agglomeration degree, which in turn depends upon the composition of the fluid, is a source of discrepancies among studies [52]. Furthermore, other parameters such as diameter, specific surface area, purity and metal contents, have been shown to influence their reactivity [53]. In particular several investigators attributed part of the observed CNT toxicity to the residual metals used as catalysts in the production process. Unfortunately, a clear picture on the role of metals is difficult to be achieved since several studies were performed with MWCNT with no specification about metal impurities, or containing, although in very low amount, several metal residues [10, 22]. In our study, MWCNTg contain only iron, in great part (0.42 %) potentially bioavailable (i.e., that may dissolve in cell medium). Results obtained with BEAS-2B cells exposed to a concentration of Fe^3+^ similar to the one potentially released at the highest concentration of MWCNTg used and to Fe-depleted MWCNTg, strongly suggest that the content of iron in MWCNT samples is not an important factor for EMT induction: Fe^3+^ alone did not modify cell morphology nor the investigated epithelial markers, while Fe-depleted MWCNTg in BEAS-2B cells are able to induce changes similar to Fe-rich MWCNTg.

Chang et al. have recently reported that the EMT contributes to SWCNT-induced pulmonary fibrosis [50]. Interestingly, because several works have reported that the tube length could be a critical factor in mesothelial injury and carcinogenesis [4, 54], Wang and Chen have more recently discussed the role of MWCNT length. In the first study, only long (20–50 μm) but not short (0.5–2 μm) MWCNT were observed to cause granuloma formation, fibroblasts increase and collagen deposition in the lung of rats [10]. Chen et al. reported similar findings in mice demonstrating that long MWCNT (5–15 μm) caused heavier lung injury compared to short MWCNT (350–700 nm) and were capable of promoting pulmonary fibrosis [22]. In the present study two different fully characterized MWCNT samples have been used (Table 1) to evaluate their fibrogenic activities. The pMWCNT sample, composed by low defective long and needle-like MWCNT (length higher than 5 μm), was used as positive control. MWCNTg were produced in order to reduce the length: indeed, this sample is composed by tubes having large diameters and a short length (mean length 1.12 μm).

As a process of epithelial plasticity, EMT is achieved when epithelial cell-cell adhesions are dissolved, the actin cytoskeleton is reorganized, and cells acquire increased cell-matrix contacts and enhanced migratory and invasive capabilities [55]. During EMT, the cells lose their polarity and cell-cell contact by down-regulating the expression of E-cadherin and other components of the cell junctions such as β-catenin, essential subunit in cell-cell adhesion complexes. Concomitantly, they up-regulate the expression of cytoskeletal proteins such as fibronectin, α-SMA and vimentin, which are essential for enhanced motility and invasiveness [56]. After 96 h of incubation, sub-cytotoxic concentrations of both pMWCNT and MWCNTg samples are able to induce a significantly altered morphology of BEAS-2B cells: exposed cells appear spindle-shaped and disorganized. The shape change is accompanied, in both cases, by a significant loss of E-cadherin expression and a significant decrease of cytosolic β-catenin, that is reflective of dissociation from E-cadherin. Moreover, transformed cells acquire mesenchymal characteristics such as significant increase of vimentin, rearrangement of actin with *de novo* synthesis of α-SMA, up-regulation of fibronectin and MMPs. Cytoskeletal reorganization and changes in cell morphology were also validated by immunofluorescent staining of both pMWCNT and MWCNTg-exposed cells. Indeed, phalloidin-labelled β-actin is predominantly organized in cortical bundles tightly associated with cell-cell adhesions in control cells, while it is assembled into thick parallel bundles, or actin stress fibres, traversing the ventral cell surface in cells exposed to both MWCNT samples; moreover, double labelling for α-SMA and vimentin of BEAS-2B cells observed by confocal microscope shows an increase of proteins expression and their reorganization inside the cell. EMT can be induced by a number of extracellular mediators depending on the cell type involved and the precise physiological context. We have performed some experiments also on A549 cells, usually still employed in in vitro studies on toxic particulates, and we observed that MWCNT-induced EMT was not cell-specific. Indeed, EMT also occurred in A549 cells: however, it is likely that the tumoral nature of A549 cells is responsible for their resistance thus requiring a stronger exposition to MWCNT, using concentrations higher than those used for BEAS-2B cells, to obtain a significant EMT event (not shown). These data were also confirmed in vivo: our experiments, performed in mice exposed to MWCNT, showed a decreased expression of E-cadherin and conversely an increased expression of vimentin, as main markers of the occurred EMT.

Interstitial lung fibrosis is characterized by increased ECM proteins production and deposition in the lung tissue: our data show elevated levels of fibronectin - due to increased transcriptional activity - in cells exposed to MWCNT. This increased production of one of the proteins essential for ECM assembly can result in an increased production/accumulation of ECM.

Moreover, it is well known that members of MMPs could be released by stressed lung cells promoting the recruitment of inflammatory and immune cells to the site of injury, or specifically modelling cellular environment and facilitating cell invasiveness [57]. Present data show significantly increased levels of MMP-2 and MMP-9 activities in the medium of cells exposed to both MWCNT samples, thus suggesting the involvement of these fibrogenic mediators in MWCNT-induced fibrosis. Furthermore, in vivo experiments performed on C57BL/6 mice revealed a significant increased deposition of collagen in their lung interstitium, indicating the induction of fibrotic process after pulmonary exposure to both MWCNT samples, although the MWCNTg-mediated event is less strong compared to that induced by pMWCNT.

Growing evidences confirm that epithelial cells play an important role in the regulation of lung fibrosis by producing cytokines and growth factors [58]. Although Palomaki et al. reported that MWCNT induced a pro-inflammatory response in human primary macrophages [54], very recently NLRP3 inflammasome activation and dependent pro-fibrotic response was also observed in primary human bronchial epithelial cells after MWCNT exposure [17]. However, TGF-β is one of the most potent fibrogenic mediators [23, 24] and it is elevated in mice exposed to MWCNT [8, 10, 19, 22, 42, 59]. In agreement with a previous work [18], in which human BEAS-2B and A549 cells exhibited significant production of TFG-β after MWCNT treatment, also our data show that exposure to both MWCNT samples induces in BEAS-2B cells significantly elevated levels of secreted TFG-β in cell culture supernatants, in a concentration-dependent way. In vivo results corroborated one more our in vitro findings: indeed increased TGF-β levels were detected in BAL of both pMWCNT and MWCNTg-exposed mice. When cells are exposed to the highest concentration of MWCNTg (44 μg/ml), the TGF-β secretion is already significant after few hours reaching a maximum after 6 h, then slightly decreasing but keeping significant levels also after 96 h, the time period that we used to detect EMT markers, thus suggesting a continuous autocrine cell stimulation. The anti-TGF-β neutralizing antibody and TGF-β siRNA gene knockdown restored the expression levels of E-cadherin and SNAIL-1, thus confirming the TGF-β involvement in our cellular model.

Fibrosis is a disease involving complex and interrelated signalling pathways. Previous studies demonstrated that MWCNT can be inducers of lung fibrogenesis suggesting several molecular mechanisms: MWCNT can stimulate oxidative stress and mitochondrial damage in lung cells [60, 61], activate nuclear factor-κB signalling to boost inflammatory and pro-fibrogenic factors secretion [6, 8, 10, 50], and finally promote lung fibrosis through EMT via the TGF-β/Smad signalling [10, 19, 22]. To improve our knowledge about the molecular mechanisms involved in MWCNT-induced toxicity, we carried out this study investigating the Smad-independent mechanisms involved in TGF-β dependent EMT that has SNAIL-1 as a main downstream transcription factor [23, 24, 31–33]. We referred to previous works in literature reporting that TGF-β is responsible for the up-regulation of the transcription factor SNAIL-1, involved in E-cadherin gene down-regulation [31, 32], and that in addition E-cadherin repression is frequently accompanied by activation of the β-catenin signalling cascade [62]. Our data demonstrate that MWCNTg promotes EMT through the activation of TGF-β-mediated SNAIL-1 signalling pathway: SNAIL-1 accumulates in the nucleus of BEAS-2B cells after few hours of MWCNTg incubation. These events may be consequence of the rapid phosphorylation and functional inactivation of GSK-3β, an ubiquitously expressed serine/threonine kinase [34] that is crucial to stabilize SNAIL-1 [35, 36] thus contributing to the EMT regulation [32]. Indeed, we were able to detect an increased phosphorylation of GSK-3β accompanied by the simultaneous accumulation of SNAIL-1 in the nucleus of cells exposed to MWCNT. The role of GSK-3β in the regulation of SNAIL-1 was confirmed by using a specific inhibitor of GSK-3β (SB216763) or siRNA gene knockdown: in the presence of these compounds, the nuclear increase of SNAIL-1 was completely prevented, and the E-cadherin expression level was partially restored.

GSK-3β is reported to be involved in many different intracellular pathways and is regulated by a number of factors: among these, we investigated PKB/Akt as an upstream regulator kinase [32, 37]. After exposure to MWCNTg the p-Akt/Akt ratio increased in a time-dependent manner reaching a peak after 2 h of incubation and then started to decrease at 4 h. Therefore, to confirm the role of Akt in GSK-3β regulation and the subsequent steps of the signalling pathway, we co-incubated BEAS-2B cells with MWCNT and a specific Akt inhibitor or siRNA gene knockdown. Interestingly, the Akt inhibitor modified neither GSK-3β phosphorylation nor SNAIL-1 accumulation in the nucleus, compared to control levels. Furthermore, also after a long-term (96 h) exposure, the Akt inhibitor or siRNA gene knockdown completely blocked the SNAIL-1 nuclear accumulation, thus recovering the expression of E-cadherin. However, the non-total recovery of the investigated epithelial marker can be explained considering TGF-β as just one of the possible mediators of MWCNT-induced EMT event in these cells.

## Conclusions

**Fig. 10:**
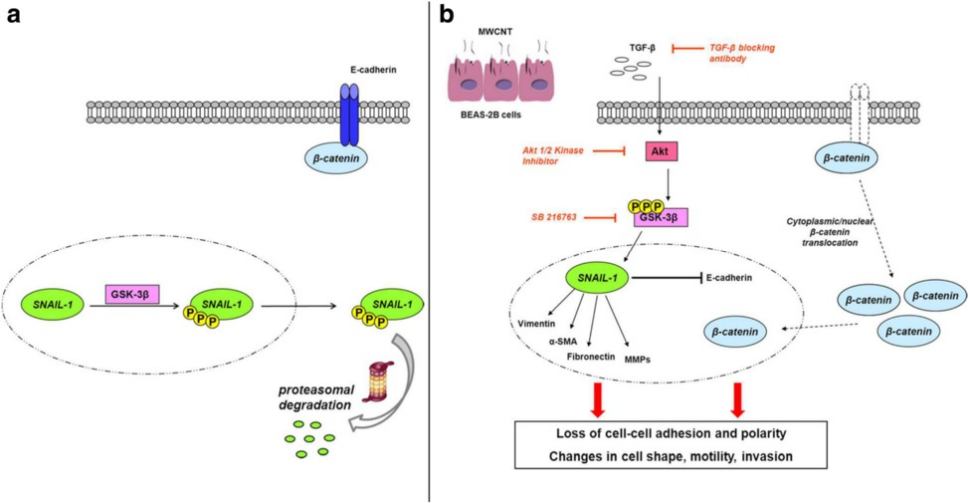
MWCNTg promote the EMT through the TGF-β-mediated Akt/GSK-3β/SNAIL-1 pathway. Normally, GSK-3β-mediated phosphorylation of nuclear SNAIL-1 mediates its subsequent degradation (**a**). MWCNTg stimulate in BEAS-2B cells TGF-β secretion, Akt activation and GSK-3β inhibition, which induces nuclear SNAIL-1 and its transcriptional activity. Contemporarily, GSK-3β inactivation stabilizes cytoplasmic β-catenin, which translocates to the nucleus contributing to keep “turned on” the EMT program (**b**)

In summary, with the present data we demonstrate that MWCNT, can induce EMT in an in vitro human model (BEAS-2B cells) after long-term incubation at sub-cytotoxic concentrations. In vitro results were confirmed by in vivo experiments in C57BL/6 mice that develop pulmonary fibrosis after MWCNT treatment. The nanotubes length and iron contained in MWCNT as impurities do not appear to play a key role in EMT induction. Finally, we suggest a new molecular mechanism of EMT involving TGF-β and its intracellular effectors Akt/GSK-3β that mediate SNAIL-1 signalling pathway, as summarized in Fig. 10. It is more likely that MWCNT exposure can exert many different cellular responses involving a great amount of molecular pathways, sometimes cross-talking each other. Interestingly, after 96 h of MWCNT samples exposure, we observed a significant increment of nuclear β-catenin – due to E-cadherin repression and following translocation to nucleus – that likely reinforces signalling for EMT promotion. There is increasing evidence for interactions between TGF-β and β-catenin pathways in EMT, particularly during development [63], while a role in fibrosis is less well established: further investigation is still required to clarify a potential crosstalk between β-catenin and SNAIL-1 signalling in the induction of EMT. MWCNT are currently the focus of intense toxicological investigations due to the raising concern on their potential negative health effects: our study may improve the scientific knowledge about MWCNT-mediated lung toxicity and suggest potential novel targets in the development of therapeutic and preventive approaches.

## Methods

### Materials

Plasticware for cell culture was provided by Falcon (Becton Dickinson, Franklin Lakes, NJ). When not otherwise specified, reagents were from Sigma-Aldrich S.r.l. (Milan, Italy).

### MWCNT samples

MWCNT were obtained from Mitsui Chemicals (Kawasaki-shi, Japan; lot #080918 01 K).

1. pMWCNT, physicochemically similar to MITSUI-7, classified as possibly carcinogenic to humans by IARC (Group 2B) [64], were used as positive control.
2. MWCNTg were produced from pMWCNT by grinding in a ball mill to reduce the length [26].
3. To remove metals, an aliquot of MWCNTg (in this study referred to as Fe-depleted MWCNTg) was suspended in 1 M HCl and the suspension was stirred at 25 °C for 10 days; after that, the Fe-depleted MWCNTg were recovered by centrifugation at 13,000 × g, washed with distilled water, and dried [26].

Physicochemical properties of different MWCNT samples are shown in Table 1. MWCNTg and Fe-depleted MWCNTg were suspended in the culture medium described below at the concentration of 1 mg/ml and sonicated on ice as previously described [26], while pMWCNT were suspended in 1.4 mg/ml endotoxin-free bovine serum albumin (BSA) at the concentration of 2.5 mg/ml and sonicated on ice as described in Vietti et al. [65]. Then all MWCNT samples were diluted into fresh medium to the final concentrations of 1, 2, 4 and 8 μg/cm^2^ (corresponding to 5.5, 11, 22 and 44 μg/ml) to perform in vitro experiments.

### MWCNT characterization

The morphology of MWCNT samples was evaluated on low agglomerated regions by TEM/SEM analysis (Additional file 1: Figure S1) and Raman spectroscopy (Additional file 2: Figure S2). The average hydrodynamic size of MWCNT samples in the culture medium was evaluated by means of DLS (Zetasizer Nano-ZS, Malvern Instruments, Worcestershire, UK). The stability of the suspensions was followed for a time period of 15 min, which is largely sufficient for a correct dosage. OM (Leica DC100 microscope; Leica Microsystems GmbH, Wetzlar, Germany) was also used to detect the presence of large aggregates.

### Cells

Human bronchial epithelial cells BEAS-2B (from American Type Culture Collection, ATCC, Manassas, VA) were cultured in RPMI-1640 medium (Gibco, Paisley, UK) with 10 % FBS (foetal bovine serum) and 1 % penicillin/streptomycin without reaching confluence, and maintained in a humidified atmosphere at 37 °C and 5 % CO_2_.

### Measurement of extracellular lactate dehydrogenase (LDH) activity

The cytotoxic effect of MWCNT was measured as leakage of LDH activity into the extracellular medium [26]. Both intracellular and extracellular enzyme activity was expressed as μmol of NADH oxidized/min/dish, then extracellular LDH activity was calculated as percentage of the total (intracellular + extracellular) LDH activity in the dish.

### Optical microscopy (OM)

Cells (0.1 × 10^6^) were incubated under the experimental conditions indicated in Results, then rinsed with PBS and examined with a Leica DC100 microscope (Leica Microsystems GmbH). For each experimental point, a minimum of five microscopic fields were examined.

### Quantitative real-time polymerase chain reaction (qRT-PCR)

Total RNA (2 μg) was reverse transcribed into cDNA using the iScript cDNA synthesis kit (Bio-Rad Laboratories, Hercules, CA) and qRT–PCR was carried out using IQ™ SYBR Green Supermix (Bio-Rad Laboratories). The same cDNA preparation was used for the quantification of human E-cadherin, vimentin, fibronectin, α-SMA and the housekeeping gene ribosomal subunit protein S14. The relative quantification of each sample was performed comparing each PCR gene product with the S14 product using the Gene Expression Macro (http://www3.bio-rad.com/LifeScience/jobs/2004/04-0684/genex.xls; Bio-Rad Laboratories). Primers used were: S14 (forward: 5′-AGGTGCAAGGAGCTGGGTAT-3′; reverse: 5′-TCCAGGGGTCTTGGTCCTATTT-3′), E-cadherin (forward: 5′-TACGCCTGGGACTCCACCTA-3′; reverse: 5′-CCAGAAACGGAGGCCTGAT-3′), α-SMA (forward: 5′-GACAATGGCTCTGGGCTCTGTAA-3′; reverse: 5′-ATGCCATGTTCTATCGGGTACTTCA-3′), fibronectin (forward: 5′-GTGCCTGGGCAACGGA-3′; reverse: 5′-CCCGACCCTGACCGAAG-3′), vimentin (forward: 5′-AGGAAATGGCTCGTCACCTTCGTGAATA-3′; reverse: 5′-GGAGTGTCGGTTGTTAAGAACTAGAGCT-3′).

### Cytosolic and nuclear extracts

Cytosolic and nuclear extracts were obtained with the Nuclear Extraction Kit (Active Motif, Vinci-Biochem, Florence, Italy), according to the manufacturer’s recommendations. The protein content was assessed with the Bradford reagent measuring the absorbance at 595 nm (37 °C) with a Synergy HT microplate reader (BioTek Instruments, Winooski, VT).

### Western blotting (WB)

Aliquots of cell supernatants (corrected for the content of cell proteins), cytosolic and nuclear extracts (10 μg) were separated by SDS-PAGE (8–10–12 %). The blots were probed with anti-human antibodies and then with horseradish peroxidase-conjugated antibodies diluted in PBS-Tween 0.1 % with Blocker Not-Fat Dry Milk 5 %. Proteins were detected by enhanced chemiluminescence (Bio-Rad Laboratories) using a ChemiDoc MP system (Bio-Rad Laboratories) with Image Lab image acquisition. Densitometric analysis of the bands was performed using ImageJ software (http://rsbweb.nih.gov/ij/). The relative quantification of each sample was performed comparing each band with glyceraldehyde 3-phosphate dehydrogenase (GAPDH) or TBP (TATA-binding protein), used as loading control for cytosolic and nuclear extracts respectively.

### Immunofluorescent staining

Cells (0.1 × 10^6^) were grown on sterile glass coverslips and incubated under the experimental conditions indicated in the Results, then rinsed with PBS, fixed with 4 % (w/v) paraformaldehyde (diluted in PBS) for 15 min. The samples were washed three times with PBS, permeabilized with 0.1 % v/v Triton-X 100 for 5 min on ice, probed with primary antibodies: mouse anti-β-catenin (1:2000) and anti-vimentin (1:5000) or rabbit anti-E-cadherin (1:2000) and anti-α-SMA (1:5000), followed by secondary TRITC (tetramethylrhodamine β-isothiocyanate)-conjugated anti-mouse (1:200) or AlexaFluor 488-conjugated anti-rabbit (1:400) antibodies. All antibodies were diluted in PBS + FBS 1 %. The slides were washed three times with PBS and once with water, mounted with 4 μl of Gel Mount Aqueous Mounting and examined with an Olympus FV300 laser scanning confocal microscope (Olympus Biosystems, Tokyo, Japan). In a parallel set of experiments, after fixation and permeabilization the cells were probed with phalloidin-tetramethylrhodamine B isothiocyanate (diluted 1:1000 in PBS) and stained with 4′,6-diamidino-2-phenylindole dihydrochloride (DAPI; diluted 1:10,000 in PBS containing 1 % FBS), to visualize actin cytoskeleton and cell nucleus respectively. The slides were washed three times with PBS and once with water, mounted with 4 μl of Gel Mount Aqueous Mounting and examined with a Leica DC100 fluorescence microscope (Leica Microsystems GmbH). For each experimental point, a minimum of five microscopic fields were examined.

### Antibodies

Primary antibodies to E-cadherin, GAPDH, fibronectin, SNAIL-1 and TBP were purchased from Santa Cruz Biotechnology Inc. (Santa Cruz, CA); primary antibody to β-catenin was from Abcam (Cambridge, UK); primary antibody to vimentin was from Sigma; primary antibody to α-SMA was from GeneTex (Irving, CA). Secondary horseradish peroxidase-conjugated anti-rabbit or anti-mouse antibodies were from goat (Bio-Rad Laboratories); AlexaFluor 488-conjugated antibody was from Millipore (Billerica, MA); TRITC-conjugated antibody was from Sigma.

### Specific inhibitors

The neutralizing anti-TGF-β antibody (5 μg/ml) was purchased by Abcam (Cambridge, UK); the GSK-3β inhibitor SB216763 and the Akt 1/2 Kinase Inhibitor were from Sigma, and were both used at a 5 μM concentration.

### siRNA transfection

Cells were plated in 35-mm-diameter Petri dishes (1.5 × 10^5^) and cultured in RPMI-1640 medium containing 10 % FBS. After 24 h, cells were washed once with fresh medium and transfected for 72 h with Akt 1/2 siRNA 50 nM (sc-43609; Santa Cruz Biotechnology Inc.), or GSK-3β siRNA 10 nM (sc-35527; Santa Cruz Biotechnology Inc.), or TGF-β siRNA 10 nM (sc-44146; Santa Cruz Biotechnology Inc.), according to manufacturer’s instructions. Cellular toxicity was assessed by measuring the extracellular LDH release. To verify the siRNA efficacy, cells were lysed and the expression of Akt and GSK-3β proteins was analyzed by WB, TGF-β gene expression by semi-quantitative PCR. Primers used for TGF-β were: (forward) 5′-GCCCTGGACACCAACTATTGC-3′, (reverse) 5′-GCTGCACTTGCAGGAGCGCAC-3′.

### Gelatin zymography

Cells were cultured in RPMI-1640 medium with 1 % FBS. Aliquots of cell supernatants (corrected for the content of cell proteins), were loaded on 8 % polyacrylamide gels containing 0.1 % gelatin under non-denaturing and non-reducing conditions to evaluate MMPs activity. Gel images were achieved by a ChemiDoc MP system with Image Lab image acquisition. Densitometric analysis of the white bands considered to reflect total enzymatic activity of MMP, was performed using ImageJ software with activity presented as relative units compared to control cells.

### ELISA

After incubation of BEAS-2B cells in the absence or presence of MWCNT, the extracellular medium was collected and centrifuged at 4 °C at 13,000 × g for 30 min. To determine the concentration of TGF-β in the supernatant, ELISA was performed according to the kit instructions (Invitrogen Corporation, Camarillo, CA). Absorbance was measured at 450 nm with a Synergy HT microplate reader. The cytokine amount was determined using a standard solution curve and corrected for the content of cell proteins. Results were finally expressed as relative fold change compared to control cells.

### Animals

For the in vivo studies, eight week-old C57BL/6 male mice were obtained from Charles River Italia (Calco, LC, Italy), and housed in the Tor Vergata Animal Technology Station under standard condition (25 °C, 50 % relative humidity) on a 12 h light/dark cycle with free access to water and laboratory animal food. All animal procedures were in compliance with the European Legislation (2010/63/EU) and have been approved by the Institutional Animal Care and Use Committee of the University of Tor Vergata.

### Pharyngeal aspiration

For the pharyngeal aspiration experiments mice of 20–22 g were anaesthetized using a mixture of Ketalar, 1 mg/mouse (Warner-Lambert, Zaventem, Belgium), and Rompun, 0.2 mg/mouse (Bayer, Leverkusen, Germany) given intraperitoneally. At least 4 animals were included in each experimental group. The procedure was performed by placing the animal supine on an inclined support with overextended head. The tongue was pulled out and 50 μl of MWCNT suspension, containing 120 μg of pMWCNT or MWCNTg, were deposited at its base. For the preparation of MWCNT suspension, pMWCNT and MWCNTg were dispersed in distilled water containing 1.4 mg/ml endotoxin-free BSA and sonicated on ice as described in Vietti et al. [65]. Control animals were treated with an equivalent volume of sterile water containing 1.4 mg/ml BSA. Pulling of the tongue induced two deep breaths that assured inhalation of the suspension. At the end of the procedure, animals were located in their cage on a heated pad and allowed to recover, before returning to their room. After 15, 30 and 60 days BAL was performed under terminal anaesthesia by cannulating the trachea and lavaging with 1 ml of ice-cold saline solution. The lavage was then centrifuged at 200 × g for 10 min at 4 °C and the supernatant was stored at −80 °C for further biochemical analysis.

### Sirius red staining

The left lobe of the mouse lungs was collected and processed for paraffin embedding following standard histological procedure. Five micron sections were stained using a solution of 1 mg/ml Sirius red (Direct Red 80) in saturated aqueous solution of picric acid (1.3 % in water). Specifically, sections were incubated in Weigert’s haematoxylin for 8 min in order to stain the nuclei, washed for 10 min in running tap water, and then transferred to the picro-sirius red solution for one hour. After two washes in acidified water, slides were dehydrated and mounted in a resinous medium. The slides were examined using a Zeiss Axioplan2 microscope and acquired though a Nikon camera. For each experimental point, a minimum of five microscopic fields were examined.

### Hydroxyproline quantitation

To measure the lung content of hydroxyproline a commercial kit was used (Chondrex Inc, Redmond, WA, USA. Catalog # 6017) following the manufacturer’s specifications. Results were finally expressed as μg of hydroxyproline per mg of lung tissue.

### Immunohistochemical analysis

Five micron thick lung sections were deparaffinized and rehydrated using several washes in xylene and decreasing concentrations of alcohol, following standard histological protocols. After bleaching of endogenous peroxidase activity using a solution of 0.3 % H_2_O_2_ in methanol, slides were incubated in TNB Blocking Buffer (0.1 M Tris-HCl, pH 7.5 0.15 M NaCl 0.5 % Blocking Reagent. Perkin Elmer) for 1 h at room temperature and then incubated with anti-E-Cadherin and anti-vimentin primary antibodies overnight at 4 °C. After three washes in TN buffer (0.1 M Tris-HCl, pH 7.5 0.15 M NaCl), sections were incubated with biotin-conjugated secondary antibodies for 1 h at room temperature. After three washes in TN buffer, slides were incubated twice with Streptavidin-HRP solution (1:100) for 30 min at room temperature, and once with 1:50 thyramide in amplification buffer for 7 min, following the manufacturer specifications. HRP activity was detected using DAB Substrate Kit (Vector laboratories). Slides were counterstained with Hematoxylin.

### Statistical analysis

All data in text and figures are provided as means ± SEM. The results were analyzed by a one-way Analysis of Variance (ANOVA) and Tukey’s post-hoc test (software: SPSS 21.0 for Windows, SPSS Inc., Chicago, IL). *p* < 0.05 was considered significant.

## Additional files

Additional file 1: TEM and SEM analysis. Due to the strong tendency to form aggregates, MWCNT samples were preventively dispersed in isopropyl alcohol and sonicated. Drops of the obtained solutions were then dropped on a TEM copper grid, covered with a lacey carbon film, or on carbon coated SEM stubs. TEM images were collected by a JEOL 3010-UHR TEM instrument operating at 300 kV and 2 k × 2 k pixel Gatan US1000CCD camera. **Figure S1.** Representative micrographs of MWCNT by TEM (A-B) and SEM (C-D) analysis. The morphology of pMWCNT (A,C) and MWCNTg (B,D) was obtained by low-resolution TEM (8000–10000×) and SEM (5000×) images on low agglomerated regions. (TIF 3725 kb)

Additional file 2: Raman Spectroscopy. Micro-Raman spectra were acquired using an integrated micro/macro Raman system, which included a Horiba Jobin Yvon HR800 microspectrometer, an Olympus BX41 microscope, and a CCD air-cooled detector. A polarized solid state Nd 80 mW laser operating at 532.11 nm was used as the excitation source. Calibration of the instruments was performed by measuring the Stokes and anti-Stokes bands and checking the position of the Si band at (520.7 cm^−1^). Each spectrum was acquired using a 100× objective, resulting in a laser beam size at the sample in the order of 2 μm. To optimize the signal-to-noise ratio, spectra were acquired using 10 scans of 10 s for each spectral region. To produce strong signals without inducing surface alteration due to the heat, a filter with optical density d) 0.6 was used. The software LabSpec 5 (Horiba Jobin Yvon) was used to analyze the spectra. **Figure S2.** Raman spectroscopy of MWCNT samples. Micro-Raman spectra of pMWCNT (red line) and MWCNTg (black line). The positions of the bands are indicated in the upper part of the figure. The band centered at 1340 cm^−1^ (D band) is associated with structural defects, while the band at 1570 cm^−1^ (G band) corresponds to the tangential in-plane stretching vibration of the carbon-carbon bonds within the graphene sheets. The ratio of the intensities of the D and G bands decreases upon grinding following the cleavage of C-C bonds that leads the introduction of defective sites. (TIF 51 kb)

Additional file 3: Figure S3. Characterization of MWCNT in cell medium. MWCNT were suspended in the culture medium at the concentration of 1 mg/ml and the suspension was sonicated in ice as described in Methods. 440 μl of the suspension were diluted at a final concentration of 44 μg/ml, incubated at 37 °C in a Petri dish and analyzed at 0, 24, 48, 72 and 96 h. (A) Variation of hydrodynamic size (solid line) and PDI (dotted line) during the time of incubation. (B) Representative DLS patterns. (TIF 121 kb)

Additional file 4: Figure S4. Effect of MWCNT on LDH release into the extracellular medium from BEAS-2B cells. A) BEAS-2B cells were incubated in either the absence (0 μg/ml, control) or presence of MWCNTg at the concentration of 5.5, 11, 22 and 44 μg/ml for 24 h (white square), 48 h (white circle), 72 h (black square) and 96 h (black circle). B) BEAS-2B cells were incubated for 96 h in either the absence (0 μg/ml, control) or presence of pMWCNT (white square) and MWCNTg (black square) at the concentration of 5.5, 11, 22 and 44 μg/ml. After incubation, the release of LDH activity was calculated as percentage of extracellular vs. total (extracellular + intracellular) LDH activity of the dish. Each measurement (*n* = 3) was performed in duplicate, and data are presented as means ± SEM. A) Vs control *p < 0.005; °*p* < 0.005. B) Vs control *p < 0.005; °*p* < 0.005. (TIF 107 kb)

Additional file 5: Figure S5. Representative microscope images of BEAS-2B cells (A-B) and relative expression of epithelial and mesenchymal markers (C). A-B) BEAS-2B cells were incubated for 96 h in either the absence (0 μg/ml, CTRL) or presence of [Fe^3+^] (released by FeCl_3_) similar to the one potentially released at the highest concentration of MWCNT used, Fe-depleted MWCNTg (44 μg/ml) and MWCNTg (44 μg/ml). After the incubation, the cells were rinsed with PBS and observed by OM (A) or fixed with paraformaldehyde and fluorescently labelled as described in Methods (B). A) Representative images are shown (10×; scale bar = 50 μm). B) Actin filaments were visualized in red, nuclei in blue. Representative images are shown (63×; scale bar = 10 μm). C). Relative expression of epithelial and mesenchymal markers were checked by WB. Relative expression of E-cadherin, β-catenin, α-SMA and vimentin proteins in BEAS-2B cells incubated in either the absence (0 μg/ml, CTRL, lane 1) or presence of [Fe^3+^] (released by FeCl_3,_ lane 2), Fe-depleted MWCNTg (44 μg/ml, lane 3) and MWCNTg (44 μg/ml, lane 4). GAPDH was used as loading control for cytosolic extracts. Each figure is representative of three experiments giving similar results. (TIF 1558 kb)
