# Supplementary figures and images for: Multi-walled carbon nanotubes directly induce epithelial-mesenchymal transition in human bronchial epithelial cells via the TGF-β-mediated Akt/GSK-3β/SNAIL-1 signalling pathway
Source: Part Fibre Toxicol. 2016 Jun 1;13:27. doi: 10.1186/s12989-016-0138-4 (PMC4890337; doi:10.1186/s12989-016-0138-4)

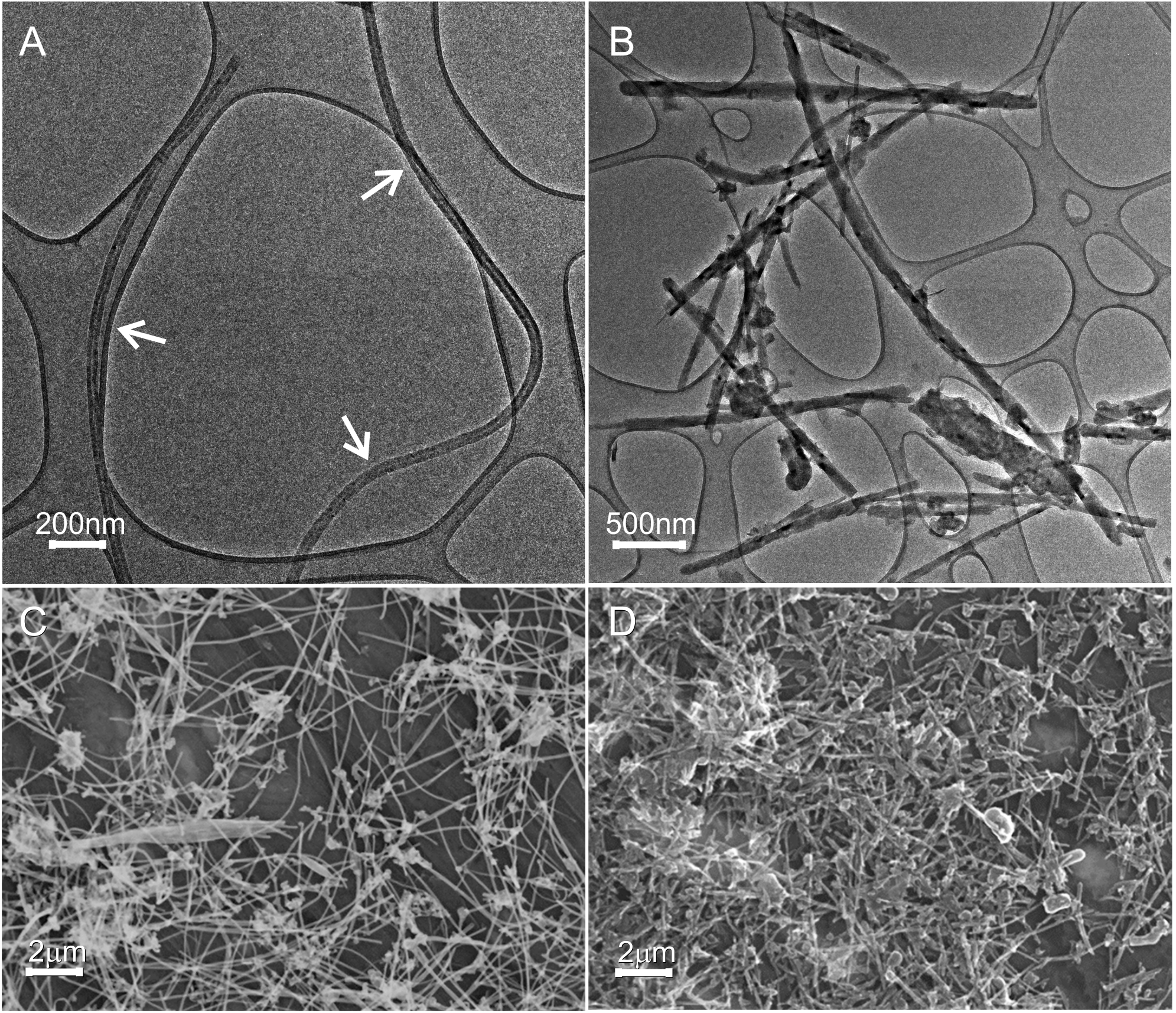

Supplement: Supplementary file 1 — TEM and SEM analysis. Due to the strong tendency to form aggregates, MWCNT samples were preventively dispersed in isopropyl alcohol and sonicated. Drops of the obtained solutions were then dropped on a TEM copper grid, covered with a lacey carbon film, or on carbon coated SEM stubs. TEM images were collected by a JEOL 3010-UHR TEM instrument operating at 300 kV and 2 k × 2 k pixel Gatan US1000CCD camera. Figure S1. Representative micrographs of MWCNT by TEM (A-B) and SEM (C-D) analysis. The morphology of pMWCNT (A,C) and MWCNTg (B,D) was obtained by low-resolution TEM (8000–10000×) and SEM (5000×) images on low agglomerated regions. (TIF 3725 kb) [file 12989_2016_138_MOESM1_ESM.tif]

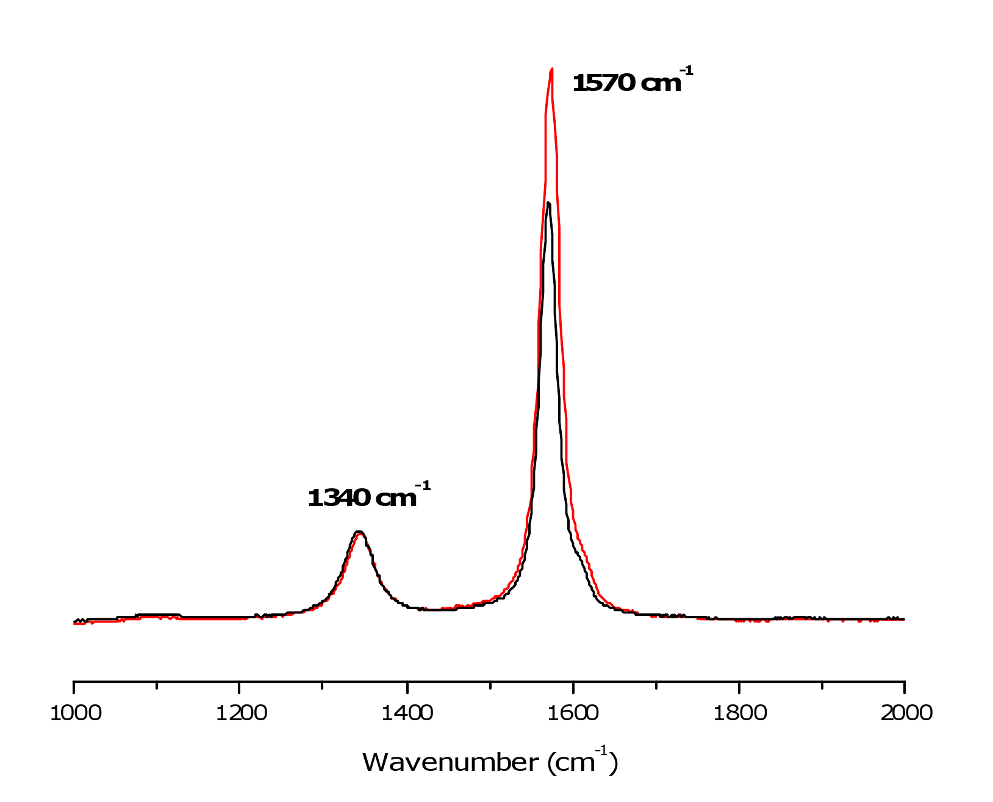

Supplement: Supplementary file 2 — Raman Spectroscopy. Micro-Raman spectra were acquired using an integrated micro/macro Raman system, which included a Horiba Jobin Yvon HR800 microspectrometer, an Olympus BX41 microscope, and a CCD air-cooled detector. A polarized solid state Nd 80 mW laser operating at 532.11 nm was used as the excitation source. Calibration of the instruments was performed by measuring the Stokes and anti-Stokes bands and checking the position of the Si band at (520.7 cm−1). Each spectrum was acquired using a 100× objective, resulting in a laser beam size at the sample in the order of 2 μm. To optimize the signal-to-noise ratio, spectra were acquired using 10 scans of 10 s for each spectral region. To produce strong signals without inducing surface alteration due to the heat, a filter with optical density d) 0.6 was used. The software LabSpec 5 (Horiba Jobin Yvon) was used to analyze the spectra. Figure S2. Raman spectroscopy of MWCNT samples. Micro-Raman spectra of pMWCNT (red line) and MWCNTg (black line). The positions of the bands are indicated in the upper part of the figure. The band centered at 1340 cm−1 (D band) is associated with structural defects, while the band at 1570 cm−1 (G band) corresponds to the tangential in-plane stretching vibration of the carbon-carbon bonds within the graphene sheets. The ratio of the intensities of the D and G bands decreases upon grinding following the cleavage of C-C bonds that leads the introduction of defective sites. (TIF 51 kb) [file 12989_2016_138_MOESM2_ESM.tif]

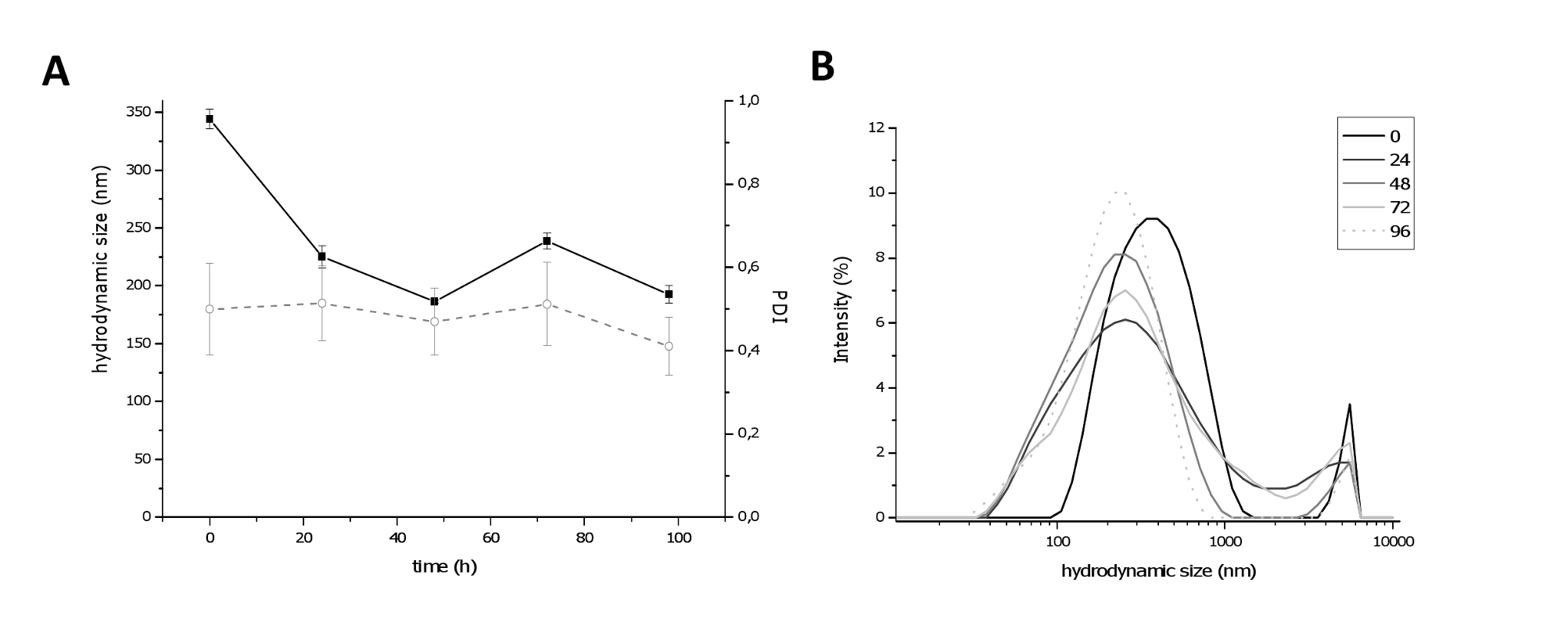

Supplement: Supplementary file 3 — Characterization of MWCNT in cell medium. MWCNT were suspended in the culture medium at the concentration of 1 mg/ml and the suspension was sonicated in ice as described in Methods. 440 μl of the suspension were diluted at a final concentration of 44 μg/ml, incubated at 37 °C in a Petri dish and analyzed at 0, 24, 48, 72 and 96 h. (A) Variation of hydrodynamic size (solid line) and PDI (dotted line) during the time of incubation. (B) Representative DLS patterns. (TIF 121 kb) [file 12989_2016_138_MOESM3_ESM.tif]

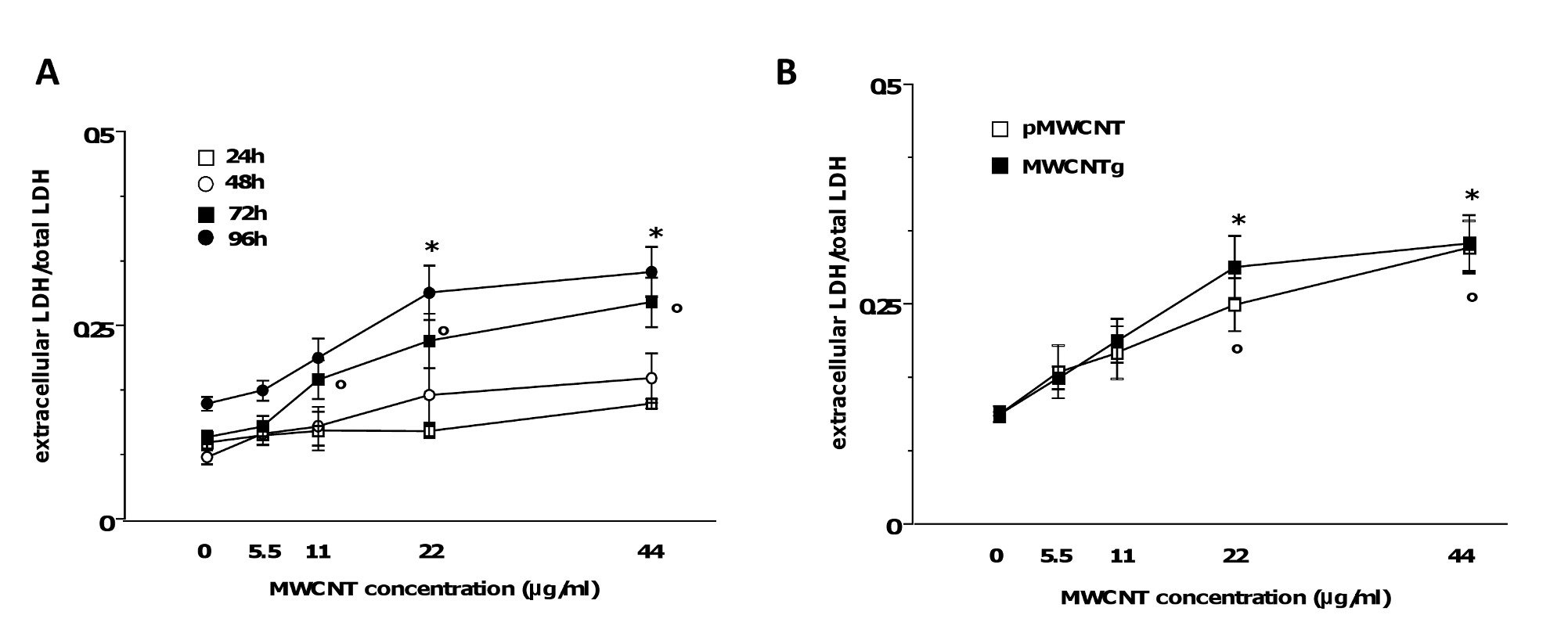

Supplement: Supplementary file 4 — Effect of MWCNT on LDH release into the extracellular medium from BEAS-2B cells. A) BEAS-2B cells were incubated in either the absence (0 μg/ml, control) or presence of MWCNTg at the concentration of 5.5, 11, 22 and 44 μg/ml for 24 h (white square), 48 h (white circle), 72 h (black square) and 96 h (black circle). B) BEAS-2B cells were incubated for 96 h in either the absence (0 μg/ml, control) or presence of pMWCNT (white square) and MWCNTg (black square) at the concentration of 5.5, 11, 22 and 44 μg/ml. After incubation, the release of LDH activity was calculated as percentage of extracellular vs. total (extracellular + intracellular) LDH activity of the dish. Each measurement (n = 3) was performed in duplicate, and data are presented as means ± SEM. A) Vs control *p < 0.005; °p < 0.005. B) Vs control *p < 0.005; °p < 0.005. (TIF 107 kb) [file 12989_2016_138_MOESM4_ESM.tif]

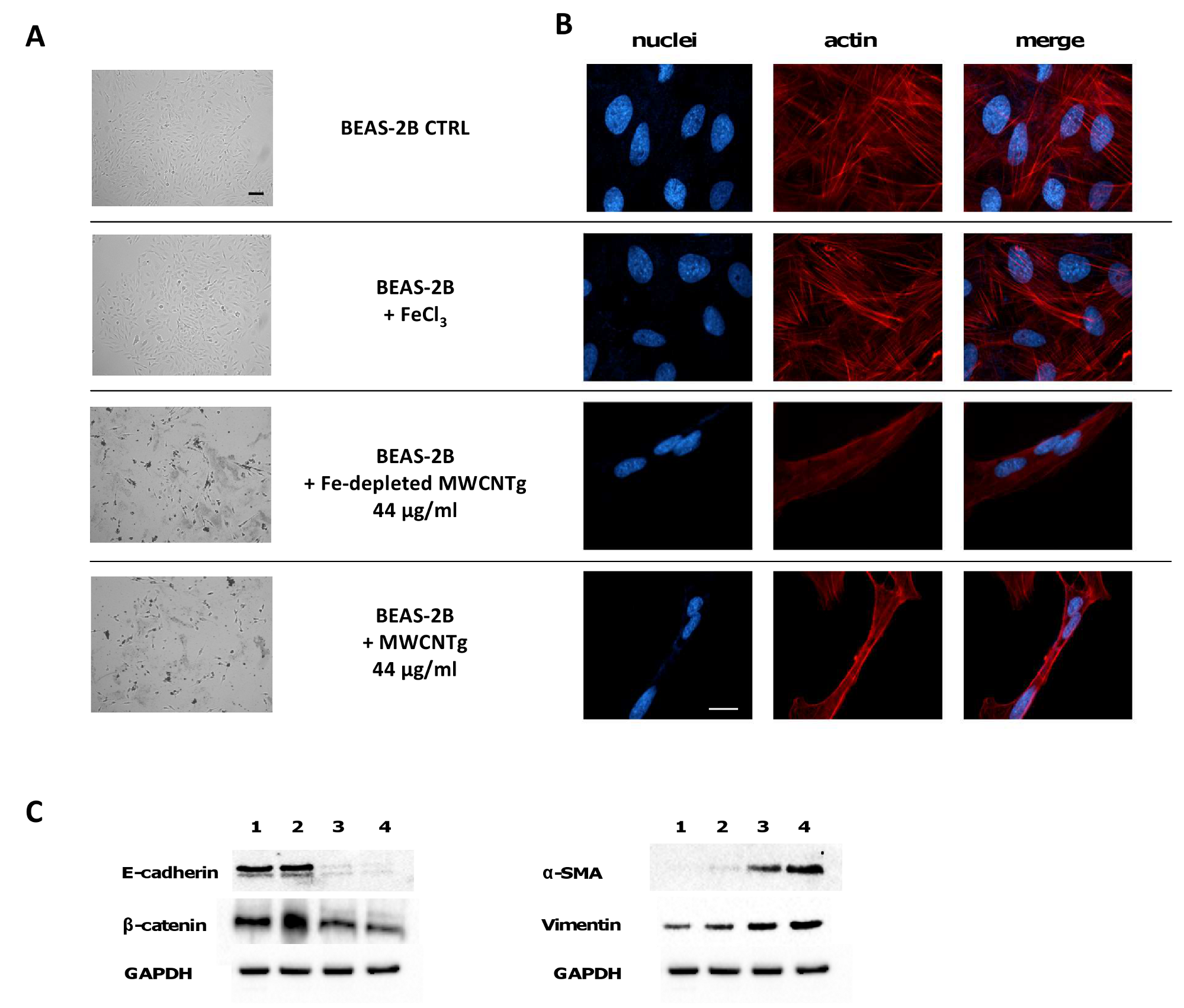

Supplement: Supplementary file 5 — Representative microscope images of BEAS-2B cells (A-B) and relative expression of epithelial and mesenchymal markers (C). A-B) BEAS-2B cells were incubated for 96 h in either the absence (0 μg/ml, CTRL) or presence of [Fe3+] (released by FeCl3) similar to the one potentially released at the highest concentration of MWCNT used, Fe-depleted MWCNTg (44 μg/ml) and MWCNTg (44 μg/ml). After the incubation, the cells were rinsed with PBS and observed by OM (A) or fixed with paraformaldehyde and fluorescently labelled as described in Methods (B). A) Representative images are shown (10×; scale bar = 50 μm). B) Actin filaments were visualized in red, nuclei in blue. Representative images are shown (63×; scale bar = 10 μm). C). Relative expression of epithelial and mesenchymal markers were checked by WB. Relative expression of E-cadherin, β-catenin, α-SMA and vimentin proteins in BEAS-2B cells incubated in either the absence (0 μg/ml, CTRL, lane 1) or presence of [Fe3+] (released by FeCl3, lane 2), Fe-depleted MWCNTg (44 μg/ml, lane 3) and MWCNTg (44 μg/ml, lane 4). GAPDH was used as loading control for cytosolic extracts. Each figure is representative of three experiments giving similar results. (TIF 1558 kb) [file 12989_2016_138_MOESM5_ESM.tif]
